# Supplementary material for: Splice-Junction-Based Mapping of Alternative Isoforms in the Human Proteome
Source: Cell Rep. Author manuscript; Available in PMC 2020 Jan 15. (PMC6961840; doi:10.1016/j.celrep.2019.11.026)
Supplement: 2 [file NIHMS1546469-supplement-2.zip › DF1/PXD006675/InferiorVenaCava/InferiorVenaCava_8_FHL1_GSSVVAYEGQSWHDYCFHCKKCSVCR.pdf]

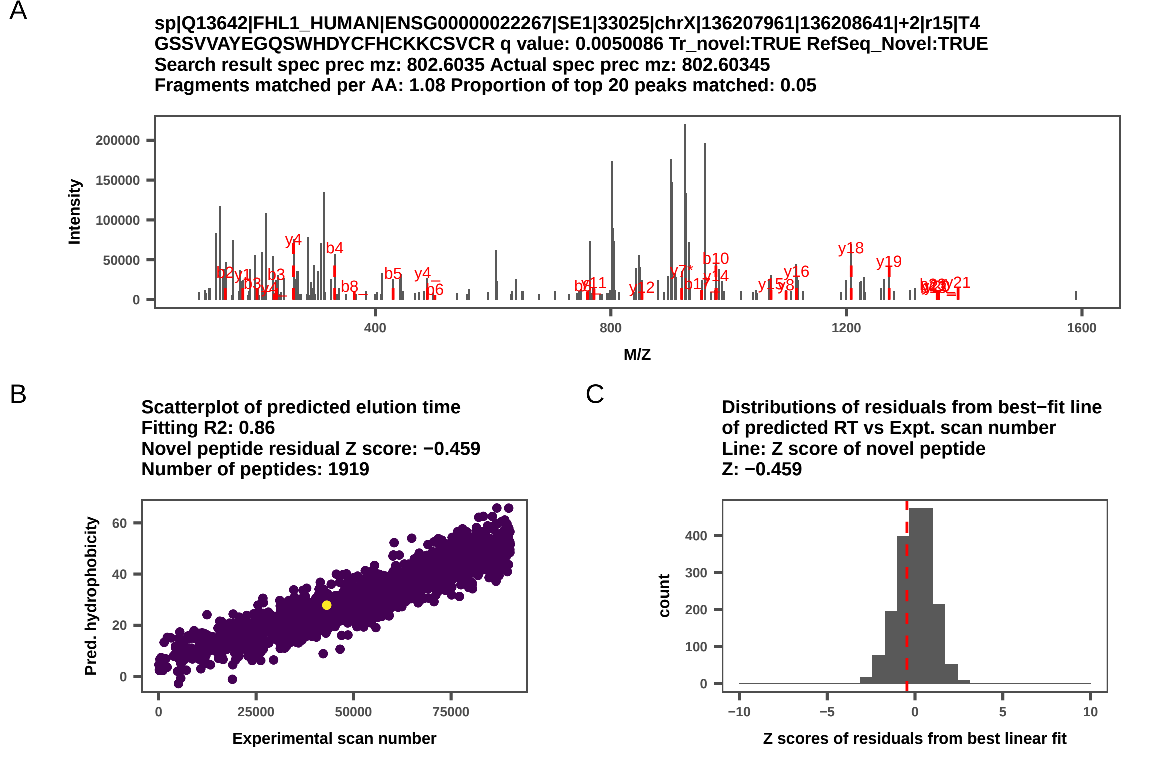
A
sp|Q13642|FHL1_HUMAN|ENSG00000022267|SE1|33025|chrX|136207961|136208641|+2|r15|T4
GSSVVAYEGQSWHDYCFHCKKCSVCR q value: 0.0050086 Tr_novel:TRUE RefSeq_Novel:TRUE
Search result spec prec mz: 802.6035 Actual spec prec mz: 802.60345
Fragments matched per AA: 1.08 Proportion of top 20 peaks matched: 0.05
Intensity
200000
150000
100000
50000
0
400
800
1200
1600
M/Z
y4
b4
b2
y1
b3
b5
b8
b6
y18
y19
y16
b10
y14
y12
y21
B
Scatterplot of predicted elution time
Fitting R2: 0.86
Novel peptide residual Z score: −0.459
Number of peptides: 1919
Pred. hydrophobicity
60
40
20
0
0
25000
50000
75000
Experimental scan number
C
Distributions of residuals from best−fit line
of predicted RT vs Expt. scan number
Line: Z score of novel peptide
Z: −0.459
count
400
300
200
100
0
−10
−5
0
5
10
Z scores of residuals from best linear fit
